# Toxicity of Some Essential Oils Constituents against Oriental Fruit Fly, *Bactrocera dorsalis* (Hendel) (Diptera: Tephritidae)

**DOI:** 10.3390/insects13100954

**Published:** 2022-10-19

**Authors:** Saleem Jaffar, Yongyue Lu

**Affiliations:** Department of Entomology, College of Plant Protection, South China Agricultural University, Guangzhou 510642, China

**Keywords:** oriental fruit fly, *B. dorsalis*, IPM, essential oils constituents, plant secondary metabolites, fumigation, ingestion toxicity, oviposition deterrence

## Abstract

**Simple Summary:**

The massive use of synthetic pesticides to manage agricultural pests results in environmental pollution and health hazards. The secondary plant metabolites, which are majorly dominated by terpenoids, have the potential to be developed into novel alternatives to synthetic chemicals. Therefore, the present study aimed at evaluating the toxicity, oviposition deterrence, and repellent activities of six majorly dominated essential oil constituents against adults and immature stages of oriental fruit flies*, Bactrocera dorsalis*. Our results highlight the potential of the selected essential oil constituents to be developed as a novel alternative to synthetic pesticides against *B. dorsalis*.

**Abstract:**

The massive use of synthetic pesticides to manage agricultural pests results in environmental pollution and health hazards. The secondary plant metabolites, which are majorly dominated by terpenoids, have the potential to be developed into novel alternatives to synthetic chemicals. Therefore, in our current investigation, six majorly dominated essential oil constituents were evaluated for their toxicity against adults and immature stages of oriental fruit flies, *Bactrocera dorsalis*, a worldwide fruit pest. The results indicated that carvacrol was the most toxic essential oil constituent (EOC) to adult flies, with LC_50_ of 19.48 mg/mL via fumigant assay, followed by thujone 75% mortality via ingestion toxicity test against adult fruit flies. Similarly, when larvae were dipped in different concentrations of EOCs, carvacrol appeared as the most toxic EOC with the lowest LC_50_ (29.12 mg/mL), followed by (−)-alpha-pinene (26.54 mg/mL) and (R)-(+)-limonene (29.12 mg/mL). In the oviposition deterrence tests, no egg was observed on oranges seedlings treated with 5% of each EOC (100% repellency). Regarding the repellency assay, a significantly higher number of flies (77%) were repelled from the Y-tube olfactometer arm containing (−)-alpha-pinene, followed by carvacrol (76%). Our results showed that the selected essential oil constituent has the potential to be developed as an alternative to synthetic pesticides against *B. dorsalis*. However, further research is required to assess the activities of these EOCs under open-field conditions.

## 1. Introduction

Oriental fruit fly *B. dorsalis* (Hendel) (Diptera: Tephritidae) an endemic to Southeast Asia is a polyphagous and well-known insect pest of various fruits and vegetables worldwide [1]. This insect pest is also considered one of the most significant quarantine insect pests. The infestation by *B. dorsalis* poses severe harmful effects to numerous horticultural crops, rendering them unfit for human consumption due to its oviposition sting and voracious larval feeding activities [2]. For the past few decades, the management of oriental fruit flies and several other economic pests has relied heavily on the application of chemical pesticides due to their effectiveness and quick response [3,4]. However, extensive and unmonitored use of chemical pesticides has caused various insect pests to develop resistance against many pesticides [5,6]. Many chemical insecticides have failed to control target insect pests, as these pests have evolved resistance to or neutralized the chemicals’ lethal effects using their physiological and ecological functions [7]. Similarly, several cases of *B. dorsalis* developing pesticide resistance have been documented in various parts of the world [8,9,10].

The aforementioned research on alternative environmentally friendly management techniques for oriental fruit flies is imperative. In this scenario, several government agencies such as the United States Food and Drug Administration (USFDA), Environmental Protection Agency (EPA), and European Union (EU), under the Integrated Pest Management (IPM) standards, also promote a significant reduction in the use of chemical pesticides while encouraging the adoption of more environmentally friendly approaches [11,12,13,14]. 

The quest to substitute synthetic insecticides with environmentally friendly alternatives such as plant extracts and essential oils (EOs) is another step towards adopting less damaging tools in IPM techniques. In several recent studies, plant essential oils (EOs) have been shown to have antifungal, antimicrobial, cytostatic, and insecticidal properties [14,15]. Essential oils are natural combinations of chemicals with low molecular weight, volatility, and lipophilic nature found in various plant families, including Asteraceae, Apiaceae, Lamiaceae, Myrtaceae, Lauraceae, and Rutaceae [15,16]. Essential oil components (EOCs) are classified into four classes according to their chemical structure: terpenes, terpenoids, phenylpropenes, and others. They may also contain a diversity of functional groups such as hydrocarbons (monoterpenes, sesquiterpenes, and aliphatic hydrocarbons); oxygenated compounds (monoterpene and sesquiterpene alcohols, aldehydes, ketones, esters, and other oxygenated compounds); and sulphur and nitrogen-containing compounds (thioesters, sulfides, isothiocyanates and nitrile) [15,16,17]. Components of essential oils exert their activities (mode of action) on pests in a neurotoxic way, for example, like gamma-aminobutyric acid (GABA), insect growth regulator (IGR), octopamine synapses (OS), impending digestive enzymes, inhibition of glutathione S-transferase (GST), cytochromes P450 (CYPs), and acetylcholinesterase (AchE) inhibitors for effective control in pre-and post-harvest agricultural techniques [18,19,20]. Moreover, various potential essential oils and their constituents belonging to terpenes and phenylpropanoids (α-pinene, β- pinene limonene and zingerone) showed effectiveness in integrated pest management (IPM), sterile insect technique (SIT), lure and kill techniques against many fruit flies such as *Ceratitis capitata*, *B. dorsalis*, *B. correcta*, *B. philippinensis*, *Anastrepha* and *Zeugodacus* genera [15,16]. 

Essential oils or their constituents (EOCs) have been widely researched as sources of environmentally acceptable insecticides against many agricultural and public health insect pests [15,17,20,21]. During the last few decades, researchers have developed a keen interest in the research and production of botanical pesticides [15]. However, our knowledge of their effectiveness against tephritid species is still limited. Several other tephritid flies (e.g., *Ceratitis capitata* and *B. oleae*) have been the subject of much insecticidal research on EO and their constituents [16,22]. Despite their economic importance, available literature information on EO activities on *B. dorsalis* is still very limited [23,24]. Consequently, this study intended to investigate the insecticidal properties of six EOCs, which are highly effective against insect vectors and other insect pests [12,15,25,26,27,28,29]. Insecticidal activities of carvacrol, (−)-α-bisabolol, thujone α, β, (R)-(+)-limonene, (−)-α-pinene, and (−)-β-pinene were tested against *B. dorsalis* immature stage and adults in laboratory conditions via fumigation, ingestion, larvicidal and pupicidal assays. Moreover, repellency and oviposition deterrence exhibited by EOCs were also determined. 

## 2. Materials and Methods

### 2.1. Essential Oil Constituents (EOCs) 

Six different EOCs were used in the current study. Analytically pure carvacrol CAS-No. 499-75-2 (purity 98%) and (−)-α-bisabolol CAS-No. 23089-26-1 (purity > 93%) were purchased from, Sigma (Sigma-Aldrich Inc., St. Louis, MO, USA). Thujone (α, β-mixture)/1-Isopropyl-4-methylbicyclo [3.1.0] hexan-3-one (α- and β- mixture) CAS-No. 1125-12-8 (purity > 70.0% GC.) was obtained from Tokyo Chemical Industry Co., Ltd. (Tokyo, Japan) (R)-(+)-limonene, CAS-No. 5989-54-8 (purity 97%) and (−)-alpha-pinene CAS-No. 7785-26-4 (purity 98%) and (−)-β-pinene CAS-No. 18172-67-3 (purity 99%) were purchased from Alfa Aesar (Shanghai, China). Analytically pure dimethyl acetone was purchased from Sinopharm Chemical Reagent Co., Ltd. (Shanghai, China). The investigated use of EOCs properties is tabulated in (Table 1).

### 2.2. Bioassays

The toxicity of EOCs on adults, larvae, and pupae of *B. dorsalis* was evaluated by topical, fumigants, and ingestion methods using a micro-applicator Eppendorf**^®^** 2231300002 (100−1000 μL). The formulations were prepared, following a modified protocol from Rizvi et al. 2018 [25]. Various concentrations of the treatments were prepared by adding 200 μL EOCs, 0.01% tween 80 in acetone and then mixed with a mini vortex shaker (vort: 2plus) according to the desired concentrations of 0.5, 1, 2, 3, 4, and 5% *w*/*v* for topical application (10 μL) and, were then prepared using the same solvent for ingestion toxicity EOCs 0.5 mL added into a sugar + water suspension, 0.5, 1.0, 2.0, 3.0, 4.0 and 5.0% (5 μL/L air) for fumigation application and the same formulation was used in mixture (synergistic) application were carvacrol, (−)-α-bisabolol, thujone (α, β-mixture), (R)-(+)-limonene, (−)-α-pinene, (−)-β-pinene with acetone at the 1:1. While in the CK (control) group, flies were treated with only 20% aqueous acetone comprising 0.1% tween 80 were applied. 

### 2.3. Bactrocera Dorsalis Colonies 

The culture of *B. dorsalis* was reared in the Department of Entomology, South China Agricultural University, Guangzhou, China. The *B. dorsalis* colonies were kept at 26 ± 1 °C and 70 ± 5% RH, with a photoperiod of 10:14 (L:D). Insects were reared following the guidelines of Hassan et al. [1]. The fly eggs were harvested from insect cages without any treatment and immersed in the muslin cloths, spreading on an artificial laboratory corn-maize-based diet. Third-instar larvae and two days-old pupae were randomly selected for the larval and pupicidal toxicity bioassays. Randomly selected adult flies (12−15 days old) were used in bioassays where adult flies were tested.

### 2.4. Fumigation Toxicity Bioassays 

Twenty adults of *B. dorsalis* of both sexes (12−15 days old) were placed in a glass jar (300 mL) and hermetically sealed with a lid. A sheet of filter paper was glued to the cap’s interior. EOCs (5 μL) in acetone at various concentrations (0.5, 1, 2, 3, 4, and 5%) were applied to the filter paper. A small layer of sterile gauze prevented the treated filter paper from direct contact with the insect. Only acetone (i.e., 100 μL/L air) was used as a control. Before the applying EOCs treatments, the filter papers were swung for 20 s to avoid direct exposure of acetone to flies. The jars were then sealed with parafilm and kept at 26 °C, 70% RH, and a 10:14 LD photoperiod. Each test was repeated three times, and mortality was recorded in each group for 24 h following the test [30]. 

### 2.5. Ingestion Toxicity Bioassays 

Following the modified protocol described by Benelli et al. [30] and Canale et al. [31], twenty mature flies (15 days old) of both sexes were placed in a 450 mL plastic container with a thin net inserted in the cap. The flies were given 200 μL of EOCs in 800 mL clean water at various doses (0.5−5 percent). The flies were given a sugar and hydrolyze yeast ratio of 1:1 in solid form. The solution was prepared by emulsifying the EOCs in water with 2.0 percent carboxymethylcellulose sodium salt (Sigma-Aldrich^®^) and 12.5% sucrose [30]. The various carrier was covered with a disc of sterile gauze, allowing the flies to feed without drowning. Plastic cups were filled with sterile cotton wool and drenched with EOCs; every box has a dish inside. Each experiment was performed three times, and mortality was assessed at 96 h.

### 2.6. Pupal Toxicity Bioassays 

The pupicidal activity of EOCs was determined by adopting the modified protocol by Zhang et al. [31], using the topical application method, and the pupae were separated by soft camel hair bush into groups of similar size and healthy. Two days-old pupae (n = 20) randomly selected from the cohort were then treated with 10 µL of each concentration of specific EOCs using an Eppendorf**^®^**micropipette (10−100 μL), and for control treatment, only acetone was applied topically. The treated and control pupae were put in the same 12 cm disposable Petri dishes with a layer of half-filled soil moisture of 70–80%. The pupae were placed in the same control conditions. After 7–10 days of EOC exposure, un-emerged or deformed treated pupae were recorded. Each treatment was replicated three times. 

### 2.7. Larval Toxicity Bioassays 

The dipping method previously described by Zhang et al. [32] was used and slightly modified to determine the toxicity of EOCs against *B. dorsalis* larvae. Each EOC was diluted to obtain different concentrations (0.5−5%) in a 10 mL glass tube. Thirty 3rd-instar larvae were immediately dipped in each dose for 1 min and transferred to the artificial rearing larval food (corn-based). For the control (CK), larvae were treated with acetone solvent only. After 48h exposure to treatment, larval mortality was recorded by touching with a soft camel hairbrush; the bioassays were replicated three times, and a total of 240 larvae per essential oil were tested.

### 2.8. Oviposition-Deterrent Activities 

To determine the oviposition deterrent activities (ODA) of essential oil constituents, the test was performed following a modified protocol from Zhang et al. [32] and Wangrawa et al. [33]. Ten gravid female *B. dorsalis* were introduced in a wooden cage (30 × 30 × 30 cm) with sugar, hydrolyzed yeast, and water *ad libitum* as food. We prepared solutions of EOCs as described above, and then each concentration was applied as one puff of spray (~125 μL) on individual fruit oranges. This mechanism was offered as a two-choice test; one orange with desire treatment and the other with acetone only, provided to *B. dorsalis adults* for oviposition. The ODA bioassay replicates three times, and the number of ovipositions was counted after 24 h of exposure. 

The Kramer and Mulla formula was used to determine the oviposition activity index (OAI). The index ranges from −1 to 1, with −1 indicating the most oviposition-deterrence effect and 1 indicating the lowest oviposition-deterrence effect [33,34]. The oviposition activity index (OAI) was calculated using the following formula:

$$OAI=\frac{NT-NC}{NT+NC}\;$$

where NT is the number (N) of eggs in treatment and NC is the number of eggs in control.

According to the oviposition activity index, three types of compounds can be identified:

I.I. No effect on oviposition OAI > 0.

II.II. Strong oviposition inhibiting substance −1 < OAI < −0.5.

III.III. Moderate oviposition-inhibiting substance −0.5 < OAI < 0

The percentage of effective repellency (ER) was calculated using the following formula:

$$ER=\frac{NC-NT}{NC}\times 100.$$

### 2.9. Repellency Test for the EOCs 

We followed the procedures of that Zaka et al. [35] and Jaleel et al. [36] used to test the repellency of different EOCs. A Y-tube olfactometer glass tube, with central and two lateral arm sizes of 20 cm × 5 cm, was used in this experiment. Sources of EOCs or control were placed in lateral arms connected with the glass chamber. To ensure delivery of odor-free air, both arms of the olfactometer obtained charcoal purified by a humidifier operating at a rate of 1.3 L/min. EOCs (0.5 μL, 3%) were placed inside the treatment glass chamber to test the attractancy or repellency. CK-acetone was placed in another chamber at the same amount. The virgin female and unmated males were individually released at the entry point of the olfactometer and allowed for 5 min toward selective responses. Each response was recorded when the fly movement into one arm was >3 cm and stayed for more than 1 min. The chamber was shuffled to the opposite side after every ten individual fly tests. The experiment was repeated three times. Furthermore, the Y-tube olfactometer and their accessory instruments were washed with 70% alcohol followed by distilled water and sterilized at 200 °C for 30 min after every investigation. Teflon tubing was used to connect spare parts. The olfactometer was set up in a wooden box that measured 100 × 80 × 80 cm in size, with a light intensity of 5000 lx and a temperature set at 27 ± 1 °C to measure the responses. Sixty mature adult male and female flies were used once for each odor and replicated three times. 

### 2.10. Data Analysis 

Mortality data obtained in fumigation, larval, pupal, and ingestion toxicity bioassays were analyzed with a general linear model followed by Tukey’s HSD test. A probability level of *p* < 0.05 was used for the significance of differences between means. The experimental mortality was corrected with Abbott’s formula (Abbott, 1925) before the calculation of lethal concentrations LC_50_ and LC_90_ with an associated 95% confidence interval (CL), and chi-squares were estimated using Probit analysis (Finney, 1978) in Minitab 7. Data obtained from olfactometer bioassays were analyzed using the chi-square test and Kruskal-Wallis one-way analysis of variance. GraphPad Prism Version 7 and Jamovi software were used to conduct the biological meaning of the statistical analysis. 

## 3. Results 

### 3.1. Fumigation Toxicity Bioassays

In fumigation assays, significant effects of EOCs (F_6,112_ = 200.08, *p* < 0.001), their concentrations (F_7,112_ = 503.01, *p* < 0.001) and the interaction (EOCs × concentrations) (F_42,112_ = 12.38, *p* < 0.001) was observed. At a concentration of 5%, maximum mortality (80%) of adult *B. dorsalis* was observed after treatment with carvacrol, and similar toxicity was observed after treatment with (−)-alpha-pinene with a 78% mortality rate. At 5% concentration, (−)-β-pinene showed 67% followed by (R)-(+)-limonene (55%) and (−)-α-bisabolol (47%). Minimum mortality (35%) at the highest concentration was observed in files fumigated with thujone and mixture. Overall, at all concentrations, mortality in *B. dorsalis* adults was higher after fumigation with carvacrol, followed by (−)-alpha-pinene. However, mortality caused by (−)-β-pinene fumigation was not significantly different from mortality caused by (−)-alpha-pinene at 1, 2, and 3% concentrations. Overall, the lowest mortality at all concentrations was observed after fumigation with thujone, followed by mixtures. Results showed that carvacrol was the most toxic essential oil constituent with the lowest LC_50_, 19.48 mg/mL, followed by (−)-alpha-pinene (27.94 mg/mL), (−)-β-pinene (29.0 mg/mL), and (R)-(+)-limonene (42.24 mg/mL). Thujone was the least toxic essential oil constituent with the highest LC_50_ (66.25 mg/mL) in the fumigation bioassay (Table 2). 

### 3.2. Ingestion Toxicity Bioassays

All EOCs investigated in this study showed insecticidal activity against adults of *B. dorsalis* when incorporated into the diet. A significant effect of the tested EOCs (F_6,112_ = 10.27, *p* < 0.001), their concentration (F_7,112_ = 391.01, *p* < 0.001) and the interaction (EOCs × concentrations) (F_42,112_ = 4.50, *p* < 0.001) were observed. Concentration-dependent mortality of adult flies was observed for all tested EOCs. Maximum mortality (75%) was observed in flies feeding on a thujone-incorporated diet followed by (R)-(+)-limonene with 65% mortality in adult flies (*p* = 0.94). The lowest mortality (45%) at 5% concentration was observed in flies feeding on a (−)-α-bisabolol-incorporated diet. However, mortality was not significantly different from mortality caused by (−)-alpha-pinene (51.6%) at the highest concentration. In contrast, at 4% concentration, the lowest mortality was observed in flies feeding on a diet containing thujone and (−)-α-bisabolol (∼38%). (R)-(+)-limonene caused maximum mortality at this concentration (55%) which was not significantly different from mortality caused by (−)-β-pinene, mixture, and (−)-alpha-pinene. At the lowest concentration (0.5%), thujone and carvacrol caused the lowest mortality (20–22%) of adult flies and no mortality by the mixture. To determine the effect of essential oil constituents on the mortality of adult flies, we also calculated lethal concentrations (LC50 and LC_90_) of each EOC using Probit analysis (Table 3). Overall, the highest toxicity was observed when flies fed on a diet containing (R)-(+)-limonene with the lowest LC_50_ (37.89 mg/mL), followed by (−)-β-pinene (37.89 mg/mL) and thujone (38.85 mg/mL). (−)-α-bisabolol was the least toxic essential oil constituent with the highest median lethal concentration (50.78 mg/mL). 

### 3.3. Pupal Toxicity Bioassays

From the two-way analysis of variance, the essential oil constituents had a significant effect on pupal mortality (F_6,112_ = 75.1, *p* < 0.001) as did concentrations (F_7,112_ = 175.8, *p* < 0.001) and their interaction (F_42,112_ = 6.20, *p* < 0.001). Maximum pupal mortality (97%) was observed at a higher concentration (5%) when pupae were treated with carvacrol and thujone (96%), followed by (R)-(+)-limonene (93%) and mixture (90%). Lower pupal mortality was observed after treatment with (−)-α-bisabolol and (−)-β-pinene at 1−5% concentrations compared to the rest of the essential oil constituents. At the lowest concentration, mortality caused by all EOCs was less than 40% and not significantly different from each other except (−)-alpha-pinene, which showed significantly higher mortality (53%) compared to the rest of the EOCs. Probit analysis results after the toxicity bioassay are shown in Table 4. Overall, (−)-alpha-pinene was the most toxic EOC with the lowest LC_50_, (11.40 mg/mL), followed by carvacrol with (14.89 mg/mL) LC_50_. Median lethal concentrations for thujone and (R)-(+)-limonene were very similar to each other (16.21 and 16.34 mg/mL), respectively. Overall, (−)-β-pinene was the last toxic essential oil constituent with a higher LC_50_ (27.62 mg/mL). 

### 3.4. Larval Toxicity Bioassays

All EOCs investigated in this study showed insecticidal activity against *B. dorsalis* larvae. A significant effect of the tested EOCs (F_6,112_ = 33.54, *p* < 0.001), their concentration (F_7,112_ = 1042, *p* < 0.001) and the interaction (EOCs × concentrations) (F_42,112_ = 7.27, *p* < 0.001) were observed. Maximum larval mortality (85%) was observed after treatment with thujone at a 5% concentration. However, it was not significantly different from mortalities after treatment with (−)-β-pinene, (R)-(+)-limonene, and carvacrol at this concentration. (−)-alpha-pinene and (−)-α-bisabolol caused significantly lower mortality (73%) compared to all other tested EOCs except the EOC mixture, which caused 58% larval mortality at 5% concentration. Larval mortality was concentration-dependent for all EOCs. Overall, carvacrol was the most toxic essential oil component with the lowest LC_50_ (26.19 mg/mL), followed by (−)-alpha-pinene (26.54 mg/mL) and (R)-(+)-limonene (29.12 mg/mL). The lowest toxicity was observed when essential oil as a mixture was applied to larvae with the highest LC_50_ (38.56 mg/mL) (Table 5). 

### 3.5. Oviposition-Deterrent Activities

Egg-laying by *B. dorsalis* was significantly reduced on the treated oranges compared to oranges treated with acetone. The inhibition of egg-laying by *B. dorsalis* depended on EOC concentrations (*p* < 0.05). The ODA gradually decreases with increasing concentrations for all the essential oils tested. All EOCs showed a stronger correlation between concentration and oviposition deterrence. At the maximum concentration, all EOCs strongly inhibited the oviposition of *B. dorsalis* gravid females. No eggs were observed on oranges treated with 5% of each EOC (*p* < 0.05) (100% repellency). However, at 4% concentration, the lowest oviposition deterrence was observed in fruits treated with (R)-(+)-limonene compared to fruits treated with all other EOCs. At the lowest concentration (0.5%), the minimum activity was observed in fruits treated with carvacrol (12.5%), followed by (R)-(+)-limonene (16.98%). Thujone and mixture showed 31−32% repellency at the lowest concentration, which was significantly less than the rest of the ECOs, which showed slightly higher repellency (37–40%) to oviposition on treated oranges. Overall, all ECOs showed very weak or moderate oviposition deterrence at 1 and 2% concentration and high oviposition deterrence at 3, 4, and 5% of each ECOs except (R)-(+)-limonene which moderate deterrence at 3% concentration (Table 6). 

### 3.6. Repellency Test

**Figure 1:**
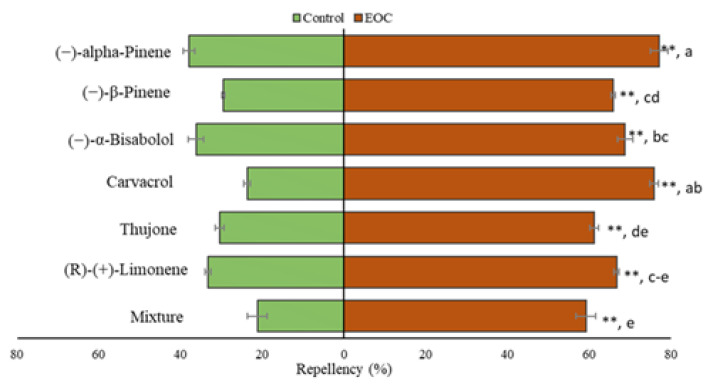
Response of *B. dorsalis* adults to the EOCs against control in a Y-tube olfactometer study. Bars marked with ‘**’ represent significantly greater repellency by EOC than control by χ^2^ analysis at *p* < 0.05. “Alphabets” on each bar represent significantly different responses between the different EOCs at the same concentration by Kruskal-Wallis H-test (at *p* < 0.05).

The responses of *B. dorsalis* in the Y-tube olfactometer at the tested concentration of EOCs are shown in (Figure 1). Essential oils constituents showed a repellency in varying degrees against *B. dorsalis* adult flies compared to control treatment in olfactometer Bioassays. All EOCs showed significantly higher repellency at the tested concentration than the control treatment determined by χ^2^ analysis at *p* < 0.05. Similarly, a significant difference in repellence was also observed among different essential oil constituents determined by the Kruskal-Wallis statistic (H = 23.55; *p* = 0.006). A higher number of flies (77%) were repelled from the Y-tube olfactometer arm containing (−)-alpha-pinene, followed by carvacrol (76%). The lowest repellence was observed in arms containing mixture (R)-(+)-limonene and thujone (59, 61, and 65%, respectively). 

## 4. Discussion 

The result of the current study implies that the EOCs could be utilized to control this pest because they were poisonous and repulsive to *B. dorsalis* to varying degrees. The toxicity and repellency of these tested EOCs have been reported against other fruit flies and insect pests in previous research [16,22]. The constituents of essential oils studied in the current study belong to several chemical classes that can be acquired from various sources. Therefore, they may have a distinct mode of action against insect pests. 

Moreover, the biological activities of essential oils are strongly influenced by the insect species studied. The findings may vary when different insect species are employed in experiments using the same essential oil. Moreover, the disparity in toxicity across the tested compounds on fruit flies could be due to the various means of EO chemical application (contact ingestion vs. topical application) and the different developmental phases of the flies studied (larvae vs. adults) [37]. 

In our fumigation, pupicidal, and larvicidal toxicity tests, carvacrol was the most toxic EOC against *B. dorsalis*. This could be due to its interaction with tyramine, a precursor of octopamine in insect tissues, including the nervous system [38]. Carvacrol also has a cytotoxic effect when absorbed and stored in the tissues [16]. The essential oil extracted from the genera Origanum, Thymus, Coridothymus, Thymbra, Satureja and Lippia are rich sources of carvacrol [39]. Fumigation activities of carvacrol against tephritid flies have not been studied previously except for *C. capitata* [40]. However, carvacrol had good fumigant efficacy against *Lycoriella ingenua* larvae, *Culex. pipiens* adults (LC_50_ = 0.26 mg/L air) and *Reticulitermes speratus* workers (LC_50_ = 0.34 mg/Petri dish) [41,42,43]. Carvacrol fumigant toxicity against adults of *Tenebrio molitor* had LC_50_ values of 5.53 and 4.77 uL/L after 24 and 48 h, respectively [44]. The larvicidal potential of carvacrol has been observed in several insect pests, including *Anopheles stephensi*, *Anopheles subpictus*, *C. quinquefasciatus*, and *C. tritaeniorhynchus* with LC_50_ values ranging from 21.15 to 27.95 μg/mL [45]. LC_50_ of carvacrol using the leaf dipping bioassay against *Pochazia shantungensis* nymphs was 56.74 mg/L [46]. Carvacrol was very toxic to *Alphitobius diaperinus* larvae at 1% concentration and caused more than 85% mortality in laboratory bioassays [47]. Contact toxicity LD_50_ values of carvacrol were 1.30–2.60 μg/fly for male and female *Drosophila suzukii* [48], 15 μg/larva against third instar *Spodopture littoralis* [49]. Carvacrol significantly reduced the adult emergence rate in *Musca domestica* in contact toxicity assays [50]. On the other hand, the fumigant and larval toxicities of essential oils and their compounds were much lower compared to phosphine against *B. tau* (LD_50_ = 0.40 μg/insect) [51] and Emamectin benzoate (LC_50_ = 0.943 mg/L) [52] or Spinetoram (*F*_4,20_ = 67.569, *p* < 0.01) against *C. capitata* [53] in positive control.

In the ingestion toxicity bioassay, (R)-(+)-limonene was the most toxic EOC, followed by (−)-β-pinene and thujone. Essential oils obtained from many plants of the family Rutaceae are good sources of limonene and other monoterpene compounds [54]. Essential oil components of limonene and pinene, though the mode of action remains primarily unknown, on the other hand, interact with the acetylcholinesterase (AchE) and octopaminergic systems (OS). The mechanism of action of *Melaleuca alternifolia* essential oil against *C. capitata* adults has also been proposed as an anti-AChE impact [16]. Our results support the previous study in which, compared to controls, essential oils from *Baccharis dracunculifolia* and *Pinus elliottii*, which contain large quantities of α-pinene and limonene, caused 58−70% mortality in *C. capitata* adults in ingestion bioassay [55]. Similarly, despite the diet, limonene was one of the most toxic EOCs that caused substantial toxicity in adult medflies, with males being more sensitive than females [40]. However, the toxicity of EOCs was less than acetamiprid (LC_50_ = 0.448 mg/L) or chlorpyrifos (LC_50_ = 0.957 mg/L) against *B. dorsalis* [52] in positive control.

Limonene was more toxic to medfly larvae as compared to α-pinene [56]. Moreover, Papanastasious et al. [40] evaluated the impact of sub-lethal doses of limonene on medflies, suggesting that, depending on the dosage, this molecule may have a hormetic-like or insecticidal effect. Essential oils containing *α*-pinene and *α*-thujone have already been revealed to be toxic to adults *Drosophila* [57] and *C. capitata* via ingestion toxicity > 50% mortality at the dose of 1.5% (*w*/*v*) and contact toxicity LD_50_ of 0.024 μL/fly after 96 h [58] and widely recognized toxic to spider mite, *Oligonychus ununguis* [59]. In addition, the adults of *M. domestica* were more susceptible to the increased contact toxicity of *Thuja occidentalis* containing (thujone and pinene) EO (LD_50_ = 33 mg fly^−1^, after 24 h of exposure) [60]. However, the toxicity of EOCs was less toxic to pupae than *Escherichia coli*-based dsRNA (87.8% 700 μL of × 200 of 3.5 × 10^8^ cells) [61] or *Metarhizium anisopliae* (LC_50_ = 1.0 × 10^10^ conidia/mL, F_4,10_  =  251.7, *p*  <  0.001) [62] in positive control.

Essential oils are responsible for the distinctive odor of plants. Therefore, essential oils or their components also act as repellents and alter insect behavior by preventing them from flying to, landing/walking on, or ovipositing on a specific source [60]. In the current study, all EOCs showed concentration-dependent antifeedant activity and were repellent to adults *B. dorsalis*. A large group of EO extracted or their components from different families have been shown to have high repellency against many arthropod species [63]. Only a few studies have examined the repellent and oviposition-deterrent properties of essential oils and their major constituents against tephritid fruit flies [16,64,65]. However, previous studies have reported the repellent, antifeedant and biological activities of tested compounds, and other IPM techniques against various insect pests [29,66,67,68,69,70,71,72]. The Y-tube olfactometer bioassay used in the current investigation demonstrated the tested chemicals’ repellant properties. However, some essential oils or their constituents’ repellent activity is typically short-lived and only works when applied freshly. Therefore, field or semi-field testing is required to determine the repellant effectiveness of these chemicals. Moreover, adding some fixative agents to retain their repellent activity warrants further studies. 

## 5. Conclusions 

This study adds to our understanding of six essential oil constituents’ insecticidal and repellent activities against larvae and adults of the oriental fruit fly, *B. dorsalis*. Regardless of the bioassay employed, the results demonstrated that all EOCs showed insecticidal and repellent activities to *B. dorsalis* in a concentration-dependent manner. To better analyze the biological activity of these chemicals, more research is needed, including field studies to test their oviposition deterrent efficacy and field trials to examine their influence on fruit infestation. The essential oil constituents have high volatility for open field application-appropriate formulations, i.e., nano-formulation, cyclodextrin, zein nanoparticles, etc., should be prepared to enhance their persistence.

## Figures and Tables

**Table 1:** List of EOCs used in the current study along with chemical structure and their sources.

CAS No	EOC Commercial Standard	Chemical Structures	Molecular Formula	Groups	Source (e.g.,)	Solubility	Toxicology	References
499-75-2	Carvacrol 98%	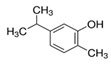	C_10_H_14_O	OM	Aromatic plants (e.g., *Origanum* spp., *Thymus* spp., *Thymbra capitata* etc.)	Ethanol, acetone, diethyl ether, carbon tetrachloride	Insecticidal activity	[25]
23089-26-1	(−)-α-Bisabolol > 93%	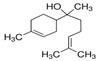	C_15_H_26_O	OS	*Matricaria chamomilla*, *Myoporum crassifolium* etc.	Slightly soluble in ethanol	Insecticidal activity	[25]
1125-12-8	Thujone (α, β-mixture) > 70.0%	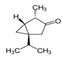	C_10_H_16_O	OMK	*Salvia officinalis, Artemisia, Tanacetum vulgare*	H_2_O at 20 °C	Neurotoxin, insecticide	[26]
5989-27-5	(R)-(+)-Limonene 97%	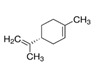	C_10_H_16_	MH	Citrus	Benzene, chloroform, ether, CS2, soluble in CCl4	Fumigant, antifeedant	[15]
7785-70-8	(−)-α-Pinene 98%	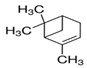	C_10_H_16_	MH	Mint, holy basil, camphor, bupleurum, and Psidium, coniferous trees	Ethanol, acetone	Insecticidal activity	[27]
18172-67-3	(−)-β-Pinene 99%	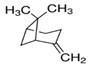	C_10_H_16_	MH	Coniferous trees and several other aromatic plants	Alcohol, chloroform, ether	Neurotoxic, insecticide	[29]

OM: Oxygenated monoterpene; OS: Oxygenated sesquiterpene; OMK: Oxygenated monoterpenoid ketone; MH: Monoterpenoid hydrocarbon.

**Table 2:** Toxicity of different EOCs in fumigant toxicity bioassay against *B. dorsalis* adults.

EOCs ^a^	LC_50_ (95% CL) ^b^	LC_90_ (95% CL) ^b^	Slope ± SE ^c^	χ^2^ (d.f.) ^d^	*p*-Value
**(−)-alpha-Pinene**	27.94 (24.96–31.20)	56.28 (50.55–64.31)	1.26 ± 0.12	28.36 (5)	0.00
**(−)-β-Pinene**	29.0 (25.66–34.25)	71.6 (61.0–83.44)	0.96 ± 0.11	20.76 (5)	0.001
**(−)-α-Bisabolol**	47.31 (40.58–58.29)	95.84 (79.09–126.47)	1.24 ± 0.004	12.64 (5)	0.02
**Carvacrol**	19.48 (15.23–23.49)	59.74 (51.47–72.75)	0.62 ± 0.003	35.77 (5)	0.00
**Thujone**	66.25 (55.16–88.03)	115.25 (92.09–163.04)	1.71 ± 0.15	4.56 (5)	0.47
**(R)-(+)-Limonene**	42.24 (37.07–49.73)	83.52 (71.09–104.21)	1.31 ± 0.12	16.13 (5)	0.006
**Mixture**	57.52 (49.30–71.87)	101.63 (85.36–137.86)	1.67 ± 0.15	8.42 (5)	0.13

a: essential oil constituents; b: lethal concentration (mg/mL) killing 50% (LC_50_) or 90% (LC_90_) of the exposed population, CL, confidence limit; c: SE, standard error d: χ^2^, chi-square; d.f., degrees of freedom.

**Table 3:** Toxicity of different EOCs in ingestion toxicity bioassay against *B. dorsalis* adults.

EOCs ^a^	LC_50_ (95% CL) ^b^	LC_90_ (95% CL) ^b^	Slope ± SE ^c^	χ^2^ (d.f.) ^d^	*p*-Value
**(−)-alpha-Pinene**	43.65 (38.11–51.92)	86.61 (73.19–109.43)	1.30 ± 0.12	14.55 (5)	0.034
**(−)-β-Pinene**	37.89 (33.24–44.24)	78.86 (67.48–97.41)	1.18 ± 0.12	11.89 (5)	0.00
**(−)-α-Bisabolol**	50.78 (43.77–62.31)	96.35 (79.98–125.87)	1.42 ± 0.13	7.75 (5)	0.17
**Carvacrol**	41.97 (35.84–51.59)	92.81 (76.35–123.02)	1.05 ± 0.11	17.33 (5)	0.004
**Thujone**	38.85 (34.38–44.89)	76.56 (66.17–93.02)	1.32 ± 0.12	20.11 (5)	0.001
**(R)-(+)-Limonene**	37.08 (33.50–41.53)	67.73 (60.18–78.83)	1.55 ± 0.13	8.77 (5)	0.11
**Mixture**	41.36 (37.74–46.02)	69.37 (61.96–80.35)	1.89 ± 0.16	19.22 (5)	0.002

a: essential oil constituents; b: lethal concentration (mg/mL) killing 50% (LC_50_) or 90% (LC_90_) of the exposed population, CL, confidence limit; c: SE, standard error d: χ^2^, chi-square; d.f., degrees of freedom.

**Table 4:** Toxicity of different EOCs against *B. dorsalis* pupa.

EOCs ^a^	LC_50_ (95% CL) ^b^	LC_90_ (95% CL) ^b^	Slope ± SE ^c^	χ^2^ (d.f.) ^d^	*p*-Value
**(−)-alpha-Pinene**	11.40 (7.50–14.50)	40.80 (36.0–47.60)	0.49 ± 0.10	40.59 (5)	0.45
**(−)-β-Pinene**	27.62 (23.85–31.94)	65.40 (56.91–78.51)	0.92 ± 0.11	16.84 (5)	0.34
**(−)-α-Bisabolol**	23.89 (20.11–27.91)	62.02 (53.23–74.52)	0.80 ± 0.10	16.22 (5)	0.33
**Carvacrol**	14.89 (12.11–17.52)	38.52 (34.5–4348)	0.71 ± 0.11	17.94 (5)	0.23
**Thujone**	16.21 (13.50–18.71)	39.29 (35.39–44.34)	0.89 ± 0.11	25.22 (5)	0.32
**(R)-(+)-Limonene**	16.34 (13.29–19.16)	43.16 (38.50–49.35)	0.77 ± 0.13	27.33 (5)	0.12
**Mixture**	16.56 (13.47–19.34)	43.88 (39.29–52.12)	0.78 ± 0.15	29.13 (5)	0.23

a: essential oil constituents; b: lethal concentration (mg/mL) killing 50% (LC_50_) or 90% (LC_90_) of the exposed population, CL, confidence limit; c: SE, standard error d: χ^2^, chi-square; d.f., degrees of freedom.

**Table 5:** Toxicity of different EOCs against *B. dorsalis* larvae.

EOCs ^a^	LC_50_ (95% CL) ^b^	LC_90_ (95% CL) ^b^	Slope ± SE ^c^	χ^2^ (d.f.) ^d^	*p*-Value
**(−)-alpha-Pinene**	26.54 (22.90–30.52)	62.81 (54.92–74.74)	0.93 ± 0.11	24.68 (5)	0.00
**(−)-β-Pinene**	31.91 (29.03–35.19)	57.69 (52.20–65.26)	1.58 ± 0.13	6.45 (5)	0.14
**(−)-α-Bisabolol**	33.12 (29.51–37.24)	69.41 (56.92–75.59)	1.33 ± 0.12	12.52 (5)	0.15
**Carvacrol**	26.19 (23.39–29.21)	53.31 (48.08–66.03)	1.23 ± 0.12	20.69 (5)	0.32
**Thujone**	31.12 (28.61–33.73)	51.77 (47.62–57.15)	1.93 ± 0.15	6.35 (5)	0.32
**(R)-(+)-Limonene**	29.12 (26.41–32.23)	54.23 (49.21–60.92)	1.49 ± 0.13	13.43 (5)	0.19
**Mixture**	38.56 (35.09–42.95)	67.56 (60.32–78.07)	1.70 ± 0.14	17.23 (5)	0.24

a: essential oil constituents; b: lethal concentration (mg/mL) killing 50% (LC_50_) or 90% (LC_90_) of the exposed population, CL, confidence limit; c: SE, standard error d: χ^2^, chi-square; d.f., degrees of freedom.

**Table 6:** The oviposition-deterrent activities of different EOCs against *B. dorsalis* adults.

EOCs	Doses											
	5%		4%		3%		2%		1%		0.5%	
	EOD (%)	OAI	ER (%)	OAI	ER (%)	OAI	ER (%)	OAI	ER (%)	OAI	ER (%)	OAI
**(−)-alpha-Pinene**	100 ^a, A^	–1	93.17 ^b, A^	–0.87	75.03 ^c, A^	–0.60	63.64 ^d, B^	–0.46	47.74 ^e, AB^	–0.31	36.75 ^f, B^	–0.22
**(−)-β-Pinene**	100 ^a, A^	–1	97.58 ^a, A^	–0.95	78.03 ^b, A^	–0.63	60.98 ^c, BC^	–0.43	46.33 ^d, B^	–0.30	36.60 ^e, B^	–0.22
**(−)-α-Bisabolol**	100 ^a, A^	–1	97.32 ^a, A^	–0.94	78.42 ^b, A^	–0.64	67.55 ^c, AB^	–0.51	54.09 ^d, A^	–0.37	40.55 ^e, A^	–0.25
**Carvacrol**	100 ^a, A^	–1	95.87 ^a, A^	–0.92	68.75 ^b, B^	–0.52	43.75 ^c, D^	–0.28	22.93 ^d, E^	–0.12	12.5 ^e, E^	–0.043
**Thujone**	100 ^a, A^	–1	94.78 ^b, A^	–0.90	84.20 ^c, A^	–0.72	73.69 ^d, A^	–0.58	39.49 ^e, C^	–0.24	31.59 ^f, C^	–0.18
**(R)-(+)-Limonene**	100 ^a, A^	–1	81.14 ^b, B^	–0.68	62.28 ^c, C^	–0.45	47.16 ^d, CD^	–0.30	26.38 ^e, D^	–0.15	16.98 ^f, D^	–0.092
**Mixture**	100 ^a, A^	–1	97.32 ^a, A^	–0.94	72.99 ^b, AB^	–0.57	54.09 ^c, C^	–0.37	40.55 ^d, C^	–0.25	32.44 ^e, C^	–0.19

Small letters = Different letters show significant differences between different concentration of EOC by Tukey HSD test (*p* ˂ 0.05). Capital letters = Different letters show significant differences between different EOCs at the same concentration level by Tukey HSD test (*p* ˂ 0.05). OAI = Oviposition Active Index; EOCs = Essential oil constituents; EOD = effective oviposition deterrent.

## Data Availability

The data used in this study would be made available by the corresponding author on request.
